# Severe intertrigo resembling acquired acrodermatitis enteropathica unmasking zinc deficiency in a patient on cabozantinib therapy

**DOI:** 10.1016/j.jdcr.2024.10.041

**Published:** 2025-02-24

**Authors:** Sakshi Chopra, Olivia Jew, Virginia Lightner

**Affiliations:** a Duke University School of Medicine, Durham, North Carolina; b Department of Dermatology, University of North Carolina Medical Center, Chapel Hill, North Carolina; c Department of Dermatology, Duke University Medical Center, Durham, North Carolina

**Keywords:** acquired acrodermatitis enteropathica, cabozantinib, hepatocellular carcinoma, intertrigo, zinc deficiency

## Introduction

Cabozantinib is a tyrosine-kinase inhibitor that treats various cancers through the inhibition of vascular endothelial growth factors 1, 2, and 3, mesenchymal epithelial transition kinase, and AXL tyrosine kinase. These receptor tyrosine kinases contribute to sustained tumor angiogenesis, and their inhibition has been shown to offer survival benefit among patients with hepatocellular carcinoma (HCC).^1^ Along with HCC, cabozantinib is also approved for use in renal cell carcinoma and differentiated thyroid cancer resistant to other vascular endothelial growth factor-directed therapies.^2^

In 2019, the Food and Drug Administration approved cabozantinib for use in patients with HCC previously treated with sorafenib, a kinase inhibitor.^3^ Multiple studies have established the efficacy of cabozantinib in treating HCC, both independently and in combination with mammalian target of rapamycin inhibitors.^4^ While cabozantinib has been shown to present with skin reactions including xerosis, palmar-plantar erythrodysesthia, scrotal erythema/ulceration, and pigmentary changes, zinc deficiency and acquired acrodermatitis enteropathica has not yet been comprehensively described.^5^^,^^6^ Here, we present a patient who developed zinc deficiency causing an intertrigo-like rash while receiving cabozantinib therapy for HCC.

## Case report

A 75-year-old man with a history of HCC refractory to tremelimumab/duravalumab, a combinatorial monoclonal antibody therapy, presented to the dermatology clinic with a bilateral, macerated rash on his inguinal creases following 1 month of cabozantinib therapy. His course of cabozantinib was complicated by nausea, vomiting, and diarrhea requiring intravenous fluid resuscitation, as well as ageusia, weakness, and malaise.

**Fig 1:**
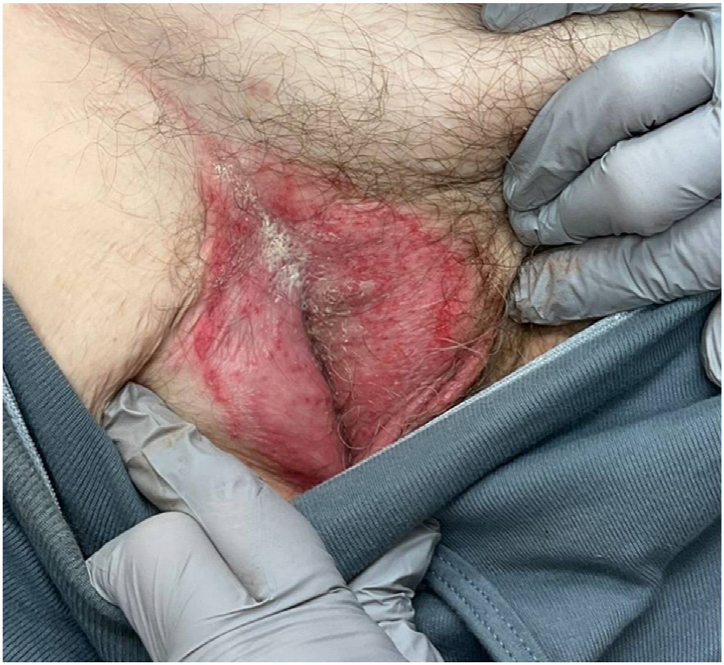
The patient presented with acrodermatitis enteropathica-like rash resembling intertrigo 1 month after starting cabozantinib.

One month after starting cabozantinib, the patient’s hands and feet developed a mild rash resembling palmar-plantar erythrodysesthesia, which was treated with petrolatum ointment. He also presented with a right scrotal rash extending to the inguinal creases, which worsened and spread after a 2-day course of nystatin powder (Fig 1). At his initial dermatology visit, candida intertrigo was suspected and he was treated with a 7-day pulse of oral fluconazole (200 mg daily) and topical miconazole cream. Limited improvement was seen after an additional 2 weeks of miconazole cream usage, and the rash ultimately failed to resolve with multiple residual lesions on the scrotum and gluteal cleft.

**Fig 2:**
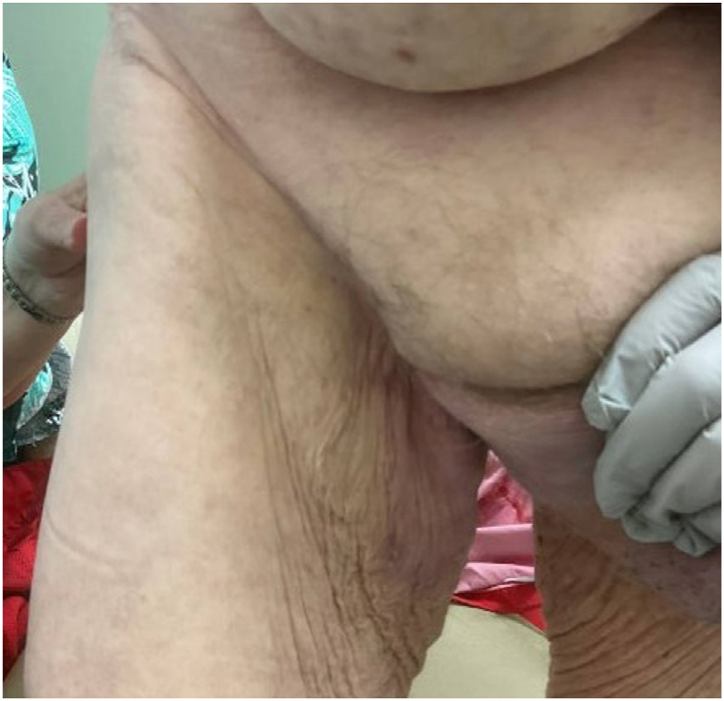
Patient presentation with near-complete resolution of inguinal rash following 2 months of treatment with zinc sulfate tablets along with pulsed oral fluconazole and miconazole cream.

Given the severity and macerated appearance of the rash, zinc deficiency was considered. Upon testing, zinc levels were found to be 46 mcg/dL (normal: 60-106 mcg/dL). After complete cessation of the antifungal regimen due to a lack of resolution, he was subsequently prescribed zinc sulfate tablets, which normalized his zinc level within 3 weeks. Within 2 months of zinc treatment, without any concurrent dermatologic or antifungal intervention, his skin symptoms nearly completely resolved (Fig 2). He regained taste, had improvement in his palmar rash, and experienced less nausea and improved strength, suggesting that zinc deficiency may have been contributing to his other symptoms as well. Unfortunately, due to gastrointestinal bleeding and deep vein thrombosis requiring anticoagulation, cabozantinib was eventually discontinued and he restarted tremelimumab/durvalumab. Zinc treatment was provided throughout, helping to prevent recurrence of the rash and his other symptoms.

## Discussion

Tyrosine kinase inhibitors such as cabozantinib have been shown to cause numerous well-documented adverse effects. These include hypertension, diarrhea, fatigue, and palmar-plantar erythrodysesthia. However, zinc deficiency, either independently or as a consequence of these other symptoms, is not an established effect of this medication class. In contrast, zinc deficiency is a documented side effect of a myriad of conditions, including postbariatric surgery, Crohn’s disease, and ulcerative colitis; in the vast majority of affected patients, correction has yielded significantly improved outcomes.^7^^,^^8^

Zinc is essential for cell production and turnover in the skin, helping to maintain a protective barrier. Zinc deficiency is an important consideration when prescribing cabozantinib because of the risk of delaying zinc-dependent processes, including anti-inflammation, wound healing, and postinjury regeneration, if it goes undiagnosed.^9^ This patient’s case of acquired zinc deficiency while on cabozantinib, with resolution of the rash once zinc levels were restored, indicates that anticancer medications like cabozantinib may somehow contribute to zinc deficiency. It is not possible to distinguish whether the etiology of this patient’s zinc deficiency was the medication itself, the physiologic impact of his cancer, or the severe malnutrition and dehydration induced by his malaise, vomiting, and diarrhea, creating an electrolyte imbalance. Even so, the rectification of many of his symptoms with zinc supplementation suggests that zinc deficiency may play a role in multiple side effects of cabozantinib and similar drugs, such as mouth ulcers, nausea, and ageusia. Given that many of the reported side effects of cabozantinib therapy overlap with symptoms of zinc deficiency, testing for zinc levels in patients with severe symptoms might allow for quick correction of an otherwise unsuspected deficiency and provide rapid relief.

Although magnesium and potassium screening are recommended with cabozantinib, routine monitoring of zinc levels is not currently recommended. This case supports a potential association between tyrosine kinase inhibitor medications like cabozantinib and zinc deficiency, albeit not necessarily a causal one. Since zinc sulfate tends to be an affordable treatment regimen, regular screening and supplementation might provide an easily accessible way to improve patients’ quality of life. In cases like the one described here, prophylactic zinc sulfate might even be directly incorporated into initial treatment for tumors like HCC to avoid inflammation, impaired healing, and other consequences of a zinc deficiency that may otherwise prove challenging to recognize and diagnose.

## Conflicts of interest

None disclosed.
